# Supplementary figures and images for: Excess burden of respiratory and abdominal conditions following COVID-19 infections during the ancestral and Delta variant periods in the United States: An EHR-based cohort study from the RECOVER program
Source: PLoS One. 2024 Jun 6;19(6):e0282451. doi: 10.1371/journal.pone.0282451 (PMC11156291; doi:10.1371/journal.pone.0282451)

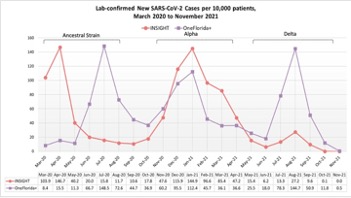

Supplement: S1 Fig — (TIFF) [file pone.0282451.s001.tiff]

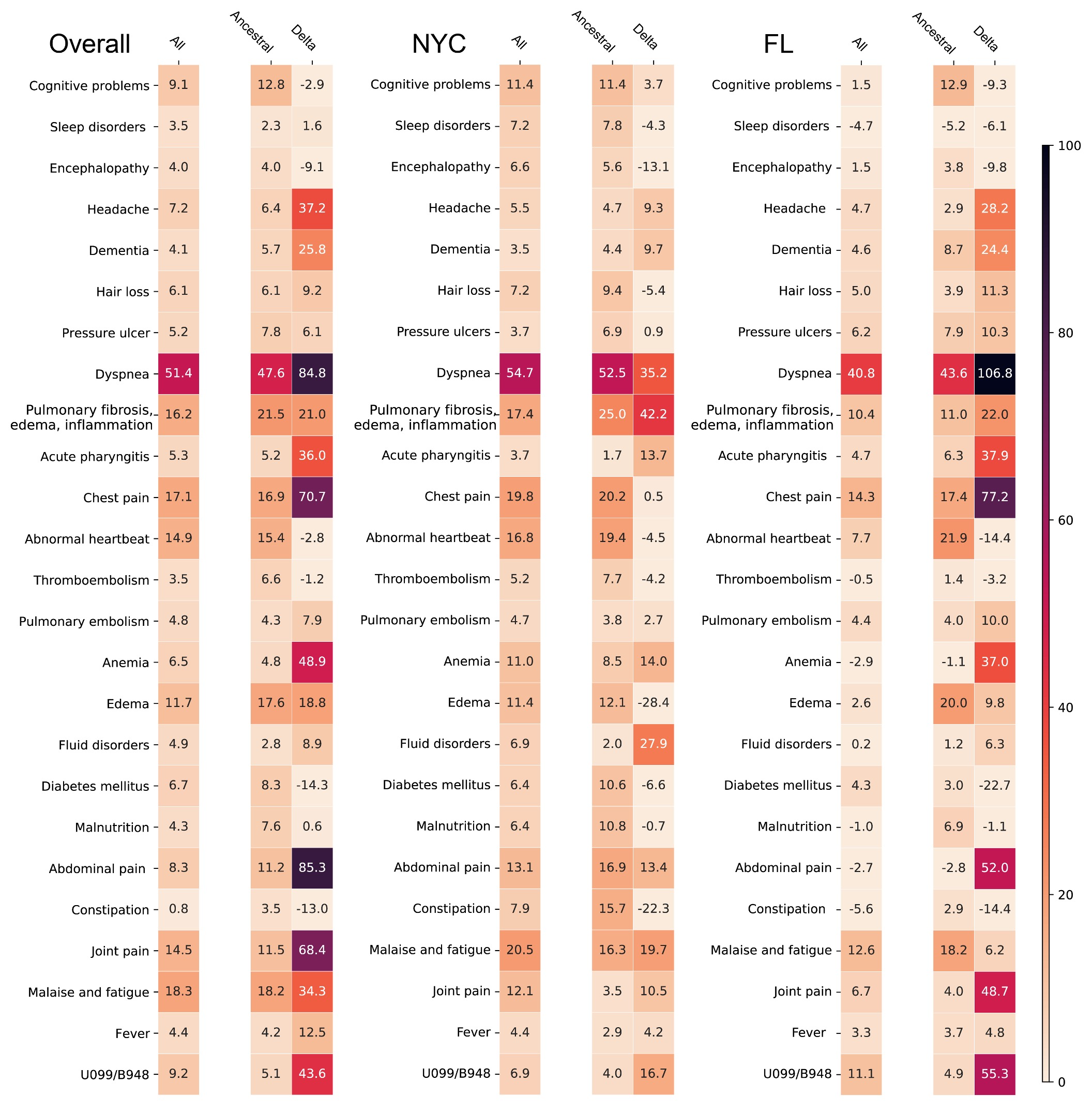

Supplement: S2 Fig — The sequelae outcomes were ascertained from day 30 after the SARS-CoV-2 infection and the adjusted hazard ratio were computed 180 days after the SARS-CoV-2 infection. (TIFF) [file pone.0282451.s002.tiff]
