# Supplementary material for: Excess burden of respiratory and abdominal conditions following COVID-19 infections during the ancestral and Delta variant periods in the United States: An EHR-based cohort study from the RECOVER program
Source: PLoS One. 2024 Jun 6;19(6):e0282451. doi: 10.1371/journal.pone.0282451 (PMC11156291; doi:10.1371/journal.pone.0282451)
Supplement: S1 Table — (DOCX) [file pone.0282451.s003.docx]

|  | **Insight** | | | **OneFlorida** | | |
| --- | --- | --- | --- | --- | --- | --- |
|  | **All** | **SARS-CoV-2 Positive** | **SARS-CoV-2 Negative** | **All** | **SARS-CoV-2 Positive** | **SARS-CoV-2 Negative** |
| **All time** | 361,401 | 35,275 | 326,126 | 199,351 | 22,341 | 177,010 |
| **Ancestral Strain Period** | 149,734 | 12,611 | 137,123 | 86,051 | 7,332 | 78,719 |
| **Delta Period** | 30,491 | 2,035 | 28,456 | 30,323 | 6,062 | 24,261 |
